# Supplementary material for: Rare variant analyses across multiethnic cohorts identify novel genes for refractive error
Source: Commun Biol. 2023 Jan 3;6:6. doi: 10.1038/s42003-022-04323-7 (PMC9810640; doi:10.1038/s42003-022-04323-7)
Supplement: Supplementary file 2 — Supplementary Information [file 42003_2022_4323_MOESM2_ESM.pdf]

Rare variant analyses across multiethnic cohorts identify novel genes for refractive error – Supplementary Materials

Anthony M. Musolf[1*], Annechien E.G Haarman[2,3*], Robert N. Luben[4,5], Jue-Sheng Ong[6], Karina Patasova[7], Rolando Hernandez Trapero[8], Joseph Marsh[8], Ishika Jain[1], Riya Jain[1], Paul Zhiping Wang[9], Deyana D. Lewis[1], Milly S. Tedja[2], Adriana I. Iglesias[2], Hengtong Li[10], Cameron S. Cowan[11], Consortium for Refractive Error and Myopia (CREAM)[†], Ginevra Biino[12], Alison P. Klein[13], Priya Duggal[14], David A. Mackey[15], Caroline Hayward[8], Toomas Haller[16], Andres Metspalu[16], Juho Wedenoja[17,18], Olavi Pärssinen[19,20], Ching-Yu Cheng[21,22], Seang-Mei Saw[23,24], Dwight Stambolian[25], Pirro G. Hysi[7], Anthony P. Khawaja[4,5], Veronique Vitart[8], Christopher J. Hammond[7], Cornelia M. van Duijn[26#], Virginie J. M. Verhoeven[2,3,27#], Caroline C. W. Klaver[2,3,11,28#], and Joan E. Bailey-Wilson[1#]

*These authors are considered co-first authors

#These authors are considered co-senior authors

†Full list and affiliations of CREAM authors are provided in the Supplement "CREAM Authors and Affiliations"

1. Computational and Statistical Genomics Branch, National Human Genome Research Institute, National Institutes of Health, Baltimore, Maryland, USA
2. Department of Ophthalmology, Erasmus Medical Center, Rotterdam, The Netherlands
3. Department of Epidemiology, Erasmus Medical Center, Rotterdam, The Netherlands
4. MRC Epidemiology, University of Cambridge School of Clinical Medicine, Cambridge, England, UK
5. NIHR Biomedical Research Centre, Moorfields Eye Hospital NHS Foundation Trust and UCL Institute of Ophthalmology, London, England, UK
6. Statistical Genetics Laboratory, Department of Genetics and Computational Biology, QIMR Berghofer Medical Research Institute, Brisbane, QLD, Australia
7. Department of Twin Research and Genetic Epidemiology, King's College London, London, England, UK
8. MRC Human Genetics Unit, Institute of Genetics and Cancer, University of Edinburgh, Western General Hospital, Edinburgh, Scotland, UK
9. Institute for Biomedical Sciences, Perelman School of Medicine, University of Pennsylvania, Philadelphia, Pennsylvania, USA
10. Data Science Unit, Singapore Eye Research Institute, Singapore National Eye Centre, Singapore, Singapore
11. Institute for Molecular and Clinical Ophthalmology Basel, Basel, Switzerland
12. Institute of Molecular Genetics, National Research Council of Italy, Pavia, Italy
13. Department of Epidemiology, Johns Hopkins University Bloomberg School of Public Health, Baltimore, Maryland, USA
14. The Bloomberg School of Public Health, Johns Hopkins University, Baltimore, Maryland, USA
15. Centre for Ophthalmology and Visual Science, Lions Eye Institute, University of Western Australia, Perth, Western Australia, Australia
16. Estonian Genome Center, Institute of Genomics, University of Tartu, Tartu, Estonia

17. Department of Ophthalmology, University of Helsinki and Helsinki University Hospital, Helsinki, Finland
18. Department of Public Health, University of Helsinki, Helsinki, Finland
19. Department of Ophthalmology, Central Hospital of Central Finland, Jyväskylä, Finland.
20. Gerontology Research Center, Faculty of Sport and Health Sciences, University of Jyväskylä, Jyväskylä, Finland.
21. Centre for Quantitative Medicine, DUKE-National University of Singapore, Singapore, Singapore
22. Ocular Epidemiology Research Group, Singapore Eye Research Institute, Singapore National Eye Centre, Singapore, Singapore
23. Saw Swee Hock School of Public Health, National University Health Systems, National University of Singapore, Singapore, Singapore
24. Myopia Research Group, Singapore Eye Research Institute, Singapore National Eye Centre, Singapore, Singapore
25. Department of Ophthalmology, University of Pennsylvania, Philadelphia, Pennsylvania, USA
26. Nuffield Department of Population Health, University of Oxford, Oxford, England, UK
27. Department of Clinical Genetics, Erasmus Medical Center, Rotterdam, The Netherlands
28. Department of Ophthalmology, Radboud University Medical Centre, Nijmegen, The Netherlands

Supplementary Note 1 – Overview of the Study Population

1. Discovery cohort studies

1.1 CREAM cohort

CREAM (Consortium for Refractive Error and Myopia) was established in 2011 as a collaboration between studies with data on refractive error which had performed genome-wide association analysis based on SNP arrays. For the current study, we included 13 participating studies with available exome chip data. Details of each study cohort and their group-specific acknowledgements are provided below.

1.2 Singapore Chinese Eye Study (SCES)

Similar to SINDI, the Singapore Chinese Eye Study (SCES) is a population-based cross-sectional study of eye diseases in Chinese adults 40 years of age or older residing in the southwestern part of Singapore. The methodology of the SCES study has been described in detail previously. Between 2009 and 2011, 3,353 (72.8%) of 4,605 eligible individuals underwent a comprehensive ophthalmologic examination, using the same protocol as SINDI.(1)

1.3 Singapore Malay Eye Study (SiMES)

SiMES is a population-based prevalence survey of Malay adults aged 40 to 79 years living in Singapore that was conducted between August of 2004 and June of 2006.(1) From a Ministry of Home Affairs random sample of 16,069 Malay adults in the Southwestern area, an age-stratified random sampling strategy was used in selecting 1,400 from each decade from age 40 years onward (40–49, 50–59, 60–69, and 70–79 years). The 4,168 eligible participants from the sampling frame, while 3280 (78.7%) participated.

Acknowledgements Singapore Malay Eye Study (SiMES): See “Acknowledgements Singapore Studies”

1.4 Singapore Indian Eye Study (SINDI)
SINDI is a population-based survey of major eye diseases(2) in ethnic Indians aged 40 to 80 years living in the South-Western part of Singapore and was conducted from August 2007 to December 2009. In brief, 4,497 Indian adults were eligible and 3,400 participated.
Acknowledgements Singapore Indian Eye Study (SINDI): See "Acknowledgements Singapore Studies"

1.5 Acknowledgements Singapore Studies: The Singapore studies (SCES, SiMES, SINDI) were supported by the National Medical Research Council, Singapore (NMRC 0796/2003, NMRC 1176/2008, STaR/0003/2008; CG/SERI/2010), Biomedical Research Council, Singapore (06/1/21/19/466, 09/1/35/19/616 and 08/1/35/19/550). The Singapore Tissue Network and the Genome Institute of Singapore, Agency for Science, Technology and Research, Singapore provided services. National supercomputing centre (NSCC) provided high performance computing resources to support GWAS analysis.

1.6 Age Related Eye Study (AREDS)
The Age-Related Eye Disease Study (AREDS) was initially designed as a long-term multicenter, prospective study of the clinical course of age-related macular degeneration (AMD) and age-related cataract(3, 4). In addition to collecting natural history data, AREDS included a randomized clinical trial of high-dose vitamin and mineral supplements for AMD and a clinical trial of high-dose vitamin supplements for cataract(3-5). Prior to study initiation, the protocol was approved by an independent data and safety monitoring committee and by the institutional review board for each clinical center. Written informed consent was obtained from all participants before enrollment in accordance with the Declaration of Helsinki. AREDS participants were 55 to 80 years of age at enrollment and had to be free of any illness or condition that would make long-term follow-up or compliance with study medications unlikely or difficult. On the basis of fundus photographs graded by a central reading center, best-corrected visual acuity and ophthalmologic evaluations, 4,757 participants were enrolled in one of several AMD categories, including persons with no AMD (control group). Visual acuity measurement of all participants was performed with the standard procedure developed for the Early Treatment of Diabetic Retinopathy Study (ETDRS). A refraction measurement was performed for participants at the randomization visit and each annual visit. For those who experience a decrease of 10 letters from baseline visual acuity, refractions were also conducted at the non-annual visits. Blood samples were collected at baseline and longitudinally as participants were send, and cell lines were established. DNA was extracted from cell lines according to standard protocols. For the current analysis, 1562 participants aged 60 and older were included from the AREDS 1a, 1b and 1c population. Refractive error measured by a refraction protocol at baseline enrollment into the AREDS study(3-6) was analyzed, taking the mean measured spherical equivalent (SE) across both eyes (or SE in a single eye when both eyes were not measured) as the trait of interest.

Acknowledgements AREDS: AREDS1a1b and FECD were supported by the National Eye Institute (grants R01EY16482, R21EY015145, and P30EY11373) and by Research to Prevent Blindness and the Ohio Lions Eye Research Foundation. The investigators gratefully acknowledge the role of the clinical coordinators and investigators who collected data on FECD cases and controls. Individual investigators and sites are listed in the first publication of the FECD study10. Data for the AREDS1a

and 1b studies was downloaded from dbGaP for analysis under a National Eye Institute data use agreement.
AREDS1c was supported by contracts from National Eye Institute/National Institutes of Health, Bethesda, MD, with additional support from Bausch & Lomb Inc, Rochester, NY. The genotyping costs were supported by the National Eye Institute (R01EY020483 to D.S.) and some of the analyses were supported by the Intramural Research Program of the National Human Genome Research Institute, National Institutes of Health, USA. AREDS acknowledges Frederick Ferris, National Eye Institute, National Institutes of Health, Bethesda, MD; and the Center for Inherited Disease Research, Baltimore, MD where SNP genotyping was carried out. The investigators gratefully acknowledge the advice and guidance of Hemin Chin of the National Eye Institute.

1.7 Rotterdam Study I (RSI)

The Rotterdam Study is a prospective population-based cohort study in the elderly living in Ommoord, a suburb of Rotterdam, the Netherlands. Details of the study are described elsewhere(7). In brief, the Rotterdam Study consists of 3 independent cohorts: RS1, RS2, and RS3. For the current analysis, 2,617 residents aged 55 years and older were included from RS1. Participants underwent multiple physical examinations with regular intervals from 1991 to present, including a nondilated automated measurement of refractive error using a Topcon RM-A2000 autorefractor. All measurements in RS-1 were conducted after the Medical Ethics Committee of the Erasmus University had approved the study protocols and all participants had given a written informed consent in accordance with the Declaration of Helsinki.

Acknowledgements Rotterdam Study and ERF: The Rotterdam Study and ERF were supported by European Research Council (ERC) under the European Union's Horizon 2020 research and innovation programme (grant 648268), Netherlands Organisation for Scientific Research (NWO, grant 91815655), Erasmus Medical Center and Erasmus University, Rotterdam, The Netherlands; Netherlands Organization for Health Research and Development (ZonMw); UitZicht; Netherlands Organisation for Scientific Research (NWO Veni 91617076 to V.J.M.V.); the Research Institute for Diseases in the Elderly; the Ministry of Education, Culture and Science; the Ministry for Health, Welfare and Sports; the European Commission (DG XII); the Municipality of Rotterdam; the Netherlands Genomics Initiative/NWO; Center for Medical Systems Biology of NGI; Lijf en Leven; M.D. Fonds; Henkes Stichting; Stichting Nederlands Oogheelkundig Onderzoek; Swart van Essen; Bevordering van Volkskracht; Blindenhulp; Landelijke Stichting voor Blinden en Slechtzienden; Rotterdamse Vereniging voor Blindenbelangen; Oogfonds; Algemene Nederlandse Vereniging ter Voorkoming van Blindheid; Stichting MaculaFonds; 17 Combined Ophthalmic Research Rotterdam; Rotterdamse Oogheelkundig Onderzoek Stichting; Erasmus MC Vriendenfonds, Topcon Europe; Novartis; Ada Hooghart, Corina Brussee, Riet Bernaerts-Biskop, Patricia van Hilten, Pascal Arp, Jeanette Vergeer, Marijn Verkerk; Sander Bervoets.

1.8 ERF

The Erasmus Rucphen Family (ERF) Study is a family-based cohort in a genetically isolated population in the southwest of the Netherlands with over 3,000 participants aged between 18 and 86 years. Crosssectional examination took place between 2002 and 2005. The rationale and study design of this study have been described elsewhere.(8, 9) Cross-sectional examination took place between 2002 and 2005, including a non-dilated automated measurement of refractive error using a Topcon RM-A2000 autorefractor. All measurements in these studies were conducted after the Medical Ethics

Committee of the Erasmus University had approved the study protocols and all participants had given a written informed consent in accordance with the Declaration of Helsinki. Acknowledgements ERF: see Rotterdam Study.

1.9 EGCUT

The Estonian cohort is from the population-based biobank of the Estonian Genome Center of the University of Tartu (EGCUT). The whole project is conducted according to the Estonian Gene Research Act and all participants have signed the broad informed consent (http://www.biobank.ee16). The current cohort size is over 51,515, from 18 years of age and up, which reflects closely the age distribution in the adult Estonian population. Subjects are recruited by the general practitioners (GP), physicians in the hospitals, and special recruitment offices of the EGCUT. They were randomly selected from the individuals visiting GP offices or hospitals. Computer Assisted Personal interviews were conducted by primary care providers and nurses during 1-2 hours at a doctor's office to collect information that includes personal data (place of birth, place(s) of living, nationality etc.), genealogical data (family history, three generations), educational and occupational history and lifestyle data (physical activity, dietary habits, smoking, alcohol consumption, women's health, quality of life) etc. Anthropometric and physiological measurements were also taken. All diseases are defined according to the ICD10 coding.(10)

Acknowledgements EGCUT: EGCUT was supported by European Union H2020 grant 692145, Est.RC grant IUT20-60 and EU Project No. 2014-2020.4.01.15-0012. They received financing by FP7 grants (201413, 245536); Estonian Government (SF0180142s08); and the European Union through the European Regional Development Fund, in the frame of Centre of Excellence in Genomics and Estonian Research Infrastructure's Roadmap; EFSD grant; and the University of Tartu (SP1GVARENG). EGCUT acknowledges Mr. T. Esko, Mr. V. Soo, Ms. M-L. Tammesoo.

1.10 FITSA

Finnish Twin Study on Aging (FITSA) is a study of genetic and environmental effects on the disablement process in older female twins.(11) The study cohort of 13 888 adult twin pairs started in 1975. Altogether 103 MZ and 114 DZ twin pairs (424 individuals, all women of European ancestry) aged 63-76 years living in Finland took part in multiple laboratory examinations in 2000 and 2003, and responded in 13 questionnaires in 2011. Before the examinations, the subjects provided a written informed consent according to the Declaration of Helsinki. The study protocol was approved by the ethics committee of the Central Hospital District of Central Finland.

Acknowledgements FITSA: FITSA was supported by ENGAGE (FP7-HEALTH-F4-2007, 201413); European Union through the GENOMEUTWIN project (QLG2-CT-2002-01254); the Academy of Finland Center of Excellence in Complex Disease Genetics (213506, 129680); the Academy of Finland Ageing Programme; and the Finnish Ministry of Culture and Education and University of Jyväskylä. For FITSA the contributions of Emmi Tikkanen, Samuli Ripatti, Markku Kauppinen, Taina Rantanen and Jaakko Kaprio are acknowledged.

1.11 Ogliastra

The Ogliastra Study is a large scale population-based epidemiological survey consisting of 12,517 inhabitants from the Ogliastra region of central eastern Sardinia, Italy from 2002 to 2008(12). The

population of Ogliastra had been genetically and socially isolated from outsiders due to its mountainous topography. Trained personnel administered a structured questionnaire collecting information on socio-demographic factors, lifestyle, drug consumption as well as medical and family history of many pathologies. This study adhered to the tenets of the Declaration of Helsinki and was approved by the institutional review board of the Italian Ministry of Education, University and Research.

Acknowledgments Ogliastra: We acknowledge the population of Ogliastra. This work was funded by a grant from the Italian Ministry of Education, University and Research (MIUR) n°: 5571/DSPAR/2002.

1.12 Croatia-Korcula

The CROATIA-Korčula study, Croatia, is a population-based, cross-sectional study in the island of Korčula that includes a total of 969 adult examinees, aged 18-98 (mean=56.3), and most (N=930) underwent a complete eye examination.(13) The study received approval from relevant ethics committees in Scotland and Croatia and followed the tenets of the Declaration of Helsinki.

Acknowledgements CROATIA-Korčula, CROATIA-Split and CROATIA-Vis studies: The CROATIA studies were funded by grants from the Medical Research Council (UK), from the Republic of Croatia Ministry of Science, Education and Sports (108-1080315-0302; 216-1080315-0302) and the Croatian Science Foundation (8875); and the CROATIA-Korčula genotyping was funded by the European Union framework program 6 project EUROSPAN (LSHGCT2006018947). The CROATIA studies acknowledges Dr. Biljana Andrijević Derk, Valentina Lacmanović Lončar, Krešimir Mandić, Antonija Mandić, Ivan Škegro, Jasna Pavičić Astaloš, Ivana Merc, Miljenka Martinović, Petra Kralj, Tamara Knežević and Katja Barać-Juretić as well as the recruitment team from the Croatian Centre for Global Health, University of Split and the Institute of Anthropological Research in Zagreb for the ophthalmological data collection; Peter Lichner and the Helmholtz Zentrum Munchen (Munich, Germany), AROS Applied Biotechnology, Aarhus, Denmark and the Wellcome Trust Clinical facility (Edinburgh, United Kingdom) for SNP array genotyping; genetic analyses were supported by the MRC HGU "QTL in Health and Disease" core program.

1.13 TwinsUK

The TwinsUK adult twin registry based at St. Thomas' Hospital in London is a volunteer cohort of over 10,000 twins from the general population.(14, 15) Twins largely volunteered unaware of the eye studies, gave fully informed consent under a protocol reviewed by the St. Thomas' Hospital Local Research Ethics Committee.

Acknowledgements TwinsUK: TwinsUK received funding from the Wellcome Trust; the European Union MyEuropia Marie Curie Research Training Network; Guide Dogs for the Blind Association; the European 18 Community's FP7 (HEALTHF22008201865GEFOS); ENGAGE (HEALTHF42007201413); the FP-5 GenomEUtwin Project (QLG2CT200201254); US National Institutes of Health/National Eye Institute (1RO1EY018246); NIH Center for Inherited Disease Research; the National Institute for Health Research comprehensive Biomedical Research Centre award to Guy's and St. Thomas' National Health Service Foundation Trust partnering with King's College London. P.G.H. is the recipient of a Fight for Sight ECI award. We acknowledge the contribution of Drs Toby Andrew, Margarida Lopes, Samantha Fahy and Diana Kozareva.

2. Replications cohort studies

1.14 The Raine Study (REHS)

The Western Australian Pregnancy Cohort Study (the Raine Study) originated as a randomized-controlled trial of 2,900 women recruited from the state's largest maternity hospital. DNA was collected from their offspring and genotyped using Human660W-Quad BeadChip (N = 1,592) and HumanOmniExpress BeadChip (N = 310). After our standard quality control and exclusion of individuals from non-Caucasian descent, genomic imputation was performed on 1,208 of the individuals genotyped with the Human660W-Quad BeadChip. A total of 1,344 of the participants completed a comprehensive eye assessment at the age of 20 years. Axial length and corneal curvature were measured with IOLMaster V.5 (Carl Zeiss Meditec AG). For axial length, five consecutive measurements were taken until the following criteria were satisfied: measurements within ± 0.02 mm of each other; good waveform, i.e., no double peaks; and acceptable signal-to-noise ratio > 2.0. Any measurement outside the mentioned criteria was deleted and repeated. During keratometry, three measurements within 0.3 D within each meridian with careful alignment and focus were recorded. Finally, a total of 1,105 individuals with genotypes (i.e., 108 genotyped and 997 imputed) and phenotypes were included in the study.

1.15 Beaver Dam eye study (BDES)

The entire details of the Beaver Dam eye study (BDES) have been previously published(16, 17). The recruitment population consisted of European ancestry individuals ages 43-84 from the town of Beaver Dam, Wisconsin, United States. 4,926 individuals were recruited into the study from 1988-1990. Baseline examinations were performed during the initial enrollment and follow-up examinations were completed every five years. Refractive error assessment was obtained through use of an automated refractor. For the approximately 4% that yielded a visual acuity of 20/40 or worse, refraction was performed using a modified Early Treatment of Diabetic Retinopathy Study (ETDRS) protocol (18). The following individuals were excluded: those with an intraocular lens in any eye, those with best corrected visual acuity of 20/200 or worse in at least one eye, and those with data missing for at least one eye. Spherical equivalent was calculated from the refraction measurements(19). Additional information such as sex, education, and familial relationships to other participants was obtained in the baseline examination. This study followed the tenets of the Declaration of Helsinki and was approved the by instutional review boards of all participating entities. All participants provided written, informed consent.

Acknowledgments BDES: We acknowledge National Institutes of Health grants EY06594(Drs R. Klein and B. E. K. Klein) and EY10605 (Dr B. E. K. Klein).

1.16 European Prospective Investigation into Cancer (EPIC-Norfolk).

The European Prospective Investigation into Cancer (EPIC) study is a pan-European prospective cohort study designed to investigate the aetiology of major chronic diseases18 . EPIC-Norfolk, one of the UK arms of EPIC, recruited and examined 25,639 participants aged 40-79 years between 1993

and 1997 for the baseline examination.(20) Recruitment was via general practices in the city of Norwich and the surrounding small towns and rural areas, and methods have been described in detail previously20 . Since virtually all residents in the UK are registered with a general practitioner through the National Health Service, general practice lists serve as population registers. Ophthalmic assessment formed part of the third health examination and this has been termed the EPIC-Norfolk Eye Study21 . In total, 8,623 participants were seen for the ophthalmic examination, between 2004 and 2011. Refractive error was measured using a Humphrey Auto-Refractor 500 (Humphrey Instruments, San Leandro, California, USA). Genotyping was undertaken using the Affymetrix GeneChip Human Mapping 500K Array Set. Data were pre-phased with SHAPEIT version 2 and imputed to the March 2012 build of the 1000 Genomes project using IMPUTE version 2.2.2. The EPIC-Norfolk Eye Study was carried out following the principles of the Declaration of Helsinki and the Research Governance Framework for Health and Social Care. The study was approved by the Norfolk Local Research Ethics Committee (05/Q0101/191) and East Norfolk & Waveney NHS Research Governance Committee (2005EC07L). All participants gave written, informed consent.
Acknowledgements EPIC-Norfolk: EPIC-Norfolk infrastructure and core functions are supported by grants from the Medical Research Council (G1000143) and Cancer Research UK (C864/A14136). The clinic for the third health examination was funded by Research into Ageing (262). Mr Khawaja is a Wellcome Trust Clinical Research Fellow. Miss Chan is a joint Medical Research Council/Royal College of Ophthalmologists Research Fellow, and received additional support from the International Glaucoma Association. Professor Foster has received additional support from the Richard Desmond Charitable Trust (via Fight for Sight) and the Department for Health through the award made by the National Institute for Health Research to Moorfields Eye Hospital and the UCL Institute of Ophthalmology for a specialist Biomedical Research Centre for Ophthalmology.

Supplementary Note 2 – Assessment of Prioritized Variants of Candidate Genes

CHST6

Carbohydrate sulfotransferase 6 (UniProt: Q9GZX3, OMIM: 605294) is a 395-amino acid protein that mediates sulfation of keratan in the cornea. Keratan sulfate plays a central role in mediating corneal transparency. When mutated, this gene is implicated in recessive macular corneal dystrophy (MCD). The variants of special interest, which occur in the lumenal domain of the protein (res. 27-395), are displayed in the figure below. These are Ala326Ser (yellow, rsid: rs201349198), Gln331His (yellow, rsid: rs140699573), Val375Gly (yellow, rsid: rs142097284), other variants from the GWAS (gray), and pathogenic ClinVar variants' positions (magenta). There was no available crystal structure to use, therefore a Phyre2 homology model (template: 3AP3, chain A) was produced for analysis. After FoldX analysis, the $\Delta\Delta G$ suggested that all three variants of special interest have only a relatively mild effect on protein stability (0.6 - 1.1 kcal/mol), compared to predicted pathogenic results for 7 of the 8 ClinVar variants (-31.03 - 7.87 kcal/mol), with the exception being a ClinVar variant that coded for the loss of a start codon (M1L: 0.22 kcal/mol). Two of the highlighted variants are positioned near known MCD-implicated variants, which suggests that the C-terminus is sensitive to variants. Overall, this information suggests that the variants could potentially interfere with keratan sulfation, which could cause a loss of function that can lead to a milder disease phenotype.

GRHL2

Grainyhead-like protein 2 homolog (UniProt: Q6ISB3, OMIM: 608576) is a 625 amino acid protein that is a transcription factor involved in primary neurulation and epithelial development. When mutated, it is implicated in autosomal dominant deafness, posterior polymorphous corneal dystrophy 4, and ectodermal dysplasia/short stature syndrome. The variants of special interest shown in yellow for this gene are Arg183Gln, Val415Ile, other GWAS variants (gray), and pathogenic ClinVar and OMIM variants' positions (magenta). Although there was a crystal structure, a homology model (template: 5MPI, chain A and 2E8M, chain A) was produced through Phyre2 due to insufficient coverage of the PDB structure (ID: 5MR7). After FoldX analysis, both variants are predicted to be relatively mild at a protein structural level, with Arg183Gln being slightly destabilizing (1.0 kcal/mol) and Val415Ile slightly stabilizing (-0.6 kcal/mol), while the already-reported variant Tyr398His (2.62 kcal/mol) is predicted to be destabilizing and Ile482Lys (0.86 kcal/mol) is slightly destabilizing.

MAPT

Microtubule-associated protein tau (UniProt: P10636, OMIM: 157140) is a 758-amino acid protein that promotes microtubule assembly and stability. When mutated, it is implicated in frontotemporal dementia, Pick disease of the brain, progressive supranuclear palsy 1, and Parkinson-dementia syndrome. The variants of special interest for this gene are shown on the structure below: Ala118Gly (yellow), Gly213Arg (yellow), Gln230Arg (yellow), Ser318Leu (yellow), Ser427Phe (yellow), Gln741Lys (yellow), other GWAS variants (gray), and the numerous pathogenic ClinVar variants' positions (magenta). There were no crystal structures with sufficient coverage for analysis, therefore a model was produced using Phyre2 for visualization purposes. While the results of the FoldX analysis using this model, which is based mostly on *de novo* modelling, are likely to be of limited reliability, it is still interesting to note that Gly213Arg (ΔΔG: 3.06 kcal/mol), which is found next to phosphoserine (res. 214), and Gln230Arg (ΔΔG: 2.51 kcal/mol) are predicted to be destabilizing. In addition, Ser318Leu is found close to Lys317Met (rsid: rs63750092), which is implicated in frontotemporal dementia with Parkinsonism, and Ser320Phe (rsid: rs63750635), which is implicated in Pick disease of the brain.

P4HTM

Transmembrane prolyl 4-hydroxylase (UniProt: Q9NXG6, OMIM: 614584) is a 502-amino acid protein that catalyses the post-translational formation of 4-hydroxyproline in hypoxia-inducible factor (HIF) alpha proteins. It hydroxylates HIF1A at Pro402 and Pro564. When mutated, it is implicated in HIDEA (hypotonia, hyperventilation, impaired intellectual development, dysautonomia, epilepsy, and eye abnormalities). The variants of special interest are shown in the structure below (PDB: 6TP5, chain A): Ile227Val (yellow), Asp386Asn (yellow), Arg273Trp (yellow), other GWAS variants in grey, and pathogenic ClinVar variants' positions (magenta). After FoldX analysis, the ΔΔG for Ile227Val (1.65 kcal/mol) is predicted to be moderately destabilizing. Ile227Val occurs in the second EF-domain (res. 224-259 in orange), occurs at a buried Ile227, and between two calcium-binding sites (res. 198-210, 237-249 in green), which could indicate functional significance for this variant, e.g., disruption of calcium binding leading to a loss of function. Asp386Asn occurs in the Fe-dependent 2-OG dioxygenase domain (res. 310-460 in red) and close to an iron-binding site at residue 374, which suggests that this variant could have an impact on iron binding, a possible introduction of a glycosylation site given its location on the protein surface or disrupt hydrogen bonds due to an asparagine substitution.

PDCD6IP

Programmed cell death 6-interacting protein (UniProt: Q9WU78, OMIM: 608074) is a 868-amino acid multifunctional protein involved in endocytosis, multivesicular body biogenesis, membrane repair, cytokinesis, apoptosis, maintenance of tight function integrity, and HIV-1 virus budding. When it is mutated, it is implicated in microcephaly. The crystal structure (PDB: 2XS1) did not provide full coverage, therefore a Phyre2 model (template: 2XS1, chain A) was produced for visualisation and energy calculations for the variant outside of range. The variants of special interest are shown in yellow in the Phyre2 structure below: Ile5Ser, Asp376Asn, Ala719Thr, Pro737Arg, and other GWAS variants (grey). After FoldX analysis, the ΔΔG for Pro737Arg (11.29 kcal/mol) and Ile5Ser (4.1 kcal/mol) showed that these variants were predicted to be highly destabilizing. Due to its predicted destabilizing effect, Pro737Arg's proximity to phosphorylation sites at Ser730 and Thr741 could be detrimental. Pro737Arg occurs in the protein's self-associating domain (res. 503-868), which could impact the domain due to the loss of the proline. Ile5Ser and Asp376Asn occurs in the protein's BRO1 domain, which is involved in endosomal targeting (res. 3-392). Furthermore, the Ile5Ser substitution could introduce a possible phosphorylation site at the N-terminus and the Asp376Asn could disrupt hydrogen bonds.

PER3

Period circadian protein homolog 3 (UniProt: P56645, OMIM: 603427) is a 1201-amino acid protein, which along with PER1 and PER2, are molecular components of the suprachiasmatic nuclei (SCN) that function as a circadian clock. When mutated, it is implicated in familial advanced sleep phase syndrome 3. The variants of special interest are shown in the I-TASSER structure (template: 4F31, chain A) below: yellow - Arg26Pro, Glu116Gly, Thr519Ala, Thr1040Asn, Ser751Asn, Arg493Cys, Ala681Gly, His416Arg, Ser919Leu, His984Tyr; grey - other GWAS variants; magenta – Pro415Ala, His417Arg. After FoldX analysis, the ΔΔG values for Arg26Pro (2.67 kcal/mol) and Thr1040Asn (-2.32 kcal/mol) are predicted to be destabilizing and stabilizing, respectively. However, since the Phyre2 prediction was based on a distant homologue, the stability predictions may not be reliable. The introduction of a disruptive proline at Arg26 is likely to be detrimental, which is reflected in the predicted ΔΔG. Ser751Asn occurs in the protein's nuclear localization signal (res. 729-745), which could disrupt hydrogen bonds. Ala681Gly occurs in the protein's CSNK1E binding domain (res. 555-760). His416Arg occurs near to the protein's nuclear export signal 3 (res. 403-412). Ser919Leu occurs near to the protein's nuclear export signal 2 (res. 925-932).

ALG3

Alpha-1,3-mannosyltransferase (UniProt: Q92685, OMIM: 608750) is a 438-amino acid protein that adds the first Dol-P-Man derived mannose in an alpha-1,3 linkage to Man5GlcNAc2-PP-Dol. When it is mutated, it is implicated in congenital disorder of glycosylation 1D (CDG1D), which can frequently present with ocular involvement and visual loss (21) . There were no available crystal structures for this protein, so a homology model was produced using Phyre2 (template: 6SNH). The variants of special interest in the structure below are Val105Phe and Val362Phe (yellow), these appear along with pathogenic ClinVar variants' positions. After FoldX analysis, the ΔΔG values for Val105Phe (8.86 kcal/mol) and Val362Phe (-2.54 kcal/mol) were predicted to be destabilizing and stabilizing, respectively. In the image below highlighting residues 80-120 and Val105 (yellow), the introduction

of a phenylalanine could disrupt the helix by interacting with other aromatic residues nearby (orange).

TNFRSF13B

TNF receptor superfamily member 13B (UniProt: O14836, OMIM: 604907) is a 293-amino acid protein that binds both TNFSF13 & TNFSF13B and is involved in the stimulation of B- and T-cell function. When it is mutated, it is implicated in immunodeficiency, common variable, 2 (CVID2) and immunoglobulin A deficiency 2 (IGAD2). There is a crystal structure (PDB: 1XU1) shown below (TNFRSF13B: sea green, TNFSF13: grey, variant position: yellow) and the FoldX energies were calculated using this structure. After FoldX analysis, Gly76Ser (5.09 kcal/mol), Gly76Cys (3.6 kcal/mol), Gly76Arg (3.26 kcal/mol) were predicted to be destabilizing. This can be due to the difference in size between a buried glycine and larger exposed serine, cysteine, and arginine are predicted by Missense3D to cause varying degrees of increase in relative surface area (RSA) for these variants (13.4%, 16.5%, 46%, respectively). The STRING interaction map shows enrichment in associations for HS-GAG or heparan sulphate – glycosaminoglycan biosynthesis (red) and heparan sulphate (purple), which could be functionally relevant due to heparan sulphate's importance in ocular disease (22).

USH2A

Usherin (UniProt: O75445, OMIM: 608400) is a 5202-amino acid protein that is involved in hearing and vision as a member of the USH2 complex. When it is mutated, it is implicated in Usher syndrome 2A and retinitis pigmentosa. Since this protein is very large, the sequence was split into 3 tracts of 1000 amino acids (res. 900-1900, 2000-3000, 3000-4000) in order to perform homology modelling using Phyre2 for visualisation and energy calculations. It is important to note that the variants of special interest shown in the images below are shown in yellow, with ClinVar variants' positions shown in magenta. For the structure covering positions 900-1900 (template: 1H30, chain A), the variants are Cys934Trp and Ser1122Pro. For the structure covering positions 2000-3000 (template: 3L5H, chain A), the variants are Pro2329His/Arg/Leu. For the structure covering positions 3000-4000 (template: 3L5H, chain A), the variant is Pro3590Gln. After FoldX analysis, the ΔΔG values for Pro2329His (99.19 kcal/mol), Ser1122Pro (23.89 kcal/mol), Pro2329Arg (20.44 kcal/mol), Pro2329Leu (15.92 kcal/mol), and Cys934Trp (3.04 kcal/mol) are predicted to be highly destabilizing. The loss of the proline for the above variants could cause an increase in conformational flexibility, which could account for the destabilization. Furthermore, a cysteine-tryptophan substitution is not favourable as there is a disulphide bond between Cys934 and Cys948. In order to investigate the significance of these highly destabilizing variants, a comparison between all USH2A ClinVar (n = 63) and gnomAD (n = 1870) variants in range was performed. After statistical testing, a significant difference was detected (Wilcoxon rank sum test – p = 0.0008) and can be seen in the boxplot below. We also compared the variants tested (shown in purple) and showed that these exhibit ΔΔG values that are much more similar to the known pathogenic variants than the putatively benign gnomAD variants.

Supplementary Figures

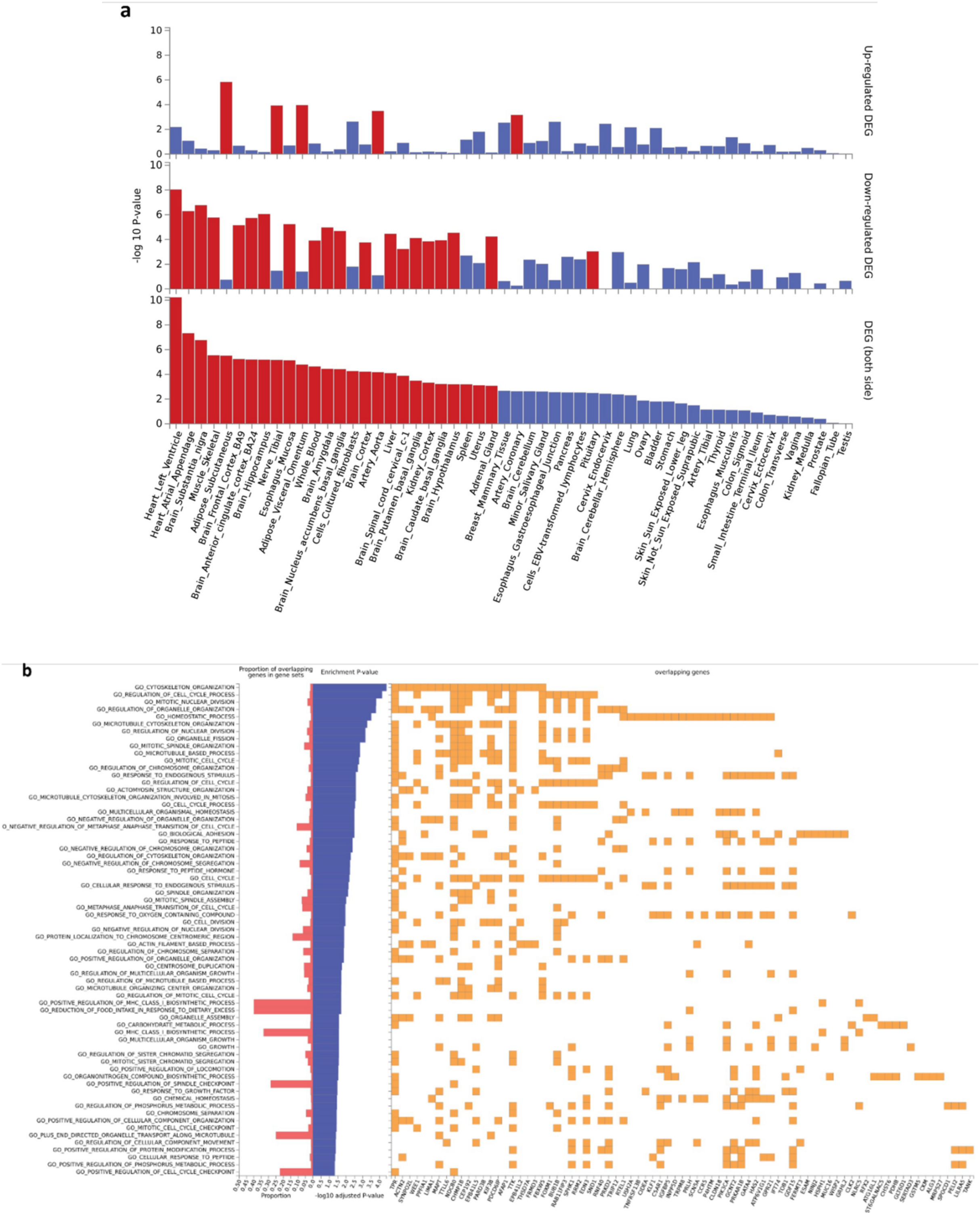

Supplementary Figure 1: Tissue expression and functional enrichment of 129 genome-wide significant genes. a) The tissue expression of the 129 genome-wide significant genes, analyzed by Functional Mapping and Annotation (FUMA). All analyzed genes were compared using GTEx v8 tissue expression data against expression data from genes from the Database of Essential Genes (DEG). The top panel shows upregulated genes with comparison to DEG; the center panel shows downregulated genes compared to DEG, and the bottom panel shows both sides. Red bars are statistically

significant. b) The overlapping functions of all 129 genome-wide significant genes, analyzed by Functional Mapping and Annotation (FUMA). Known gene function was acquired from Gene Ontology (GO). Plot contains the overlapping gene functions of the analyzed genes, the proportion of genes that overlap, the enrichment p-value, and which gene is included in a given function.

a

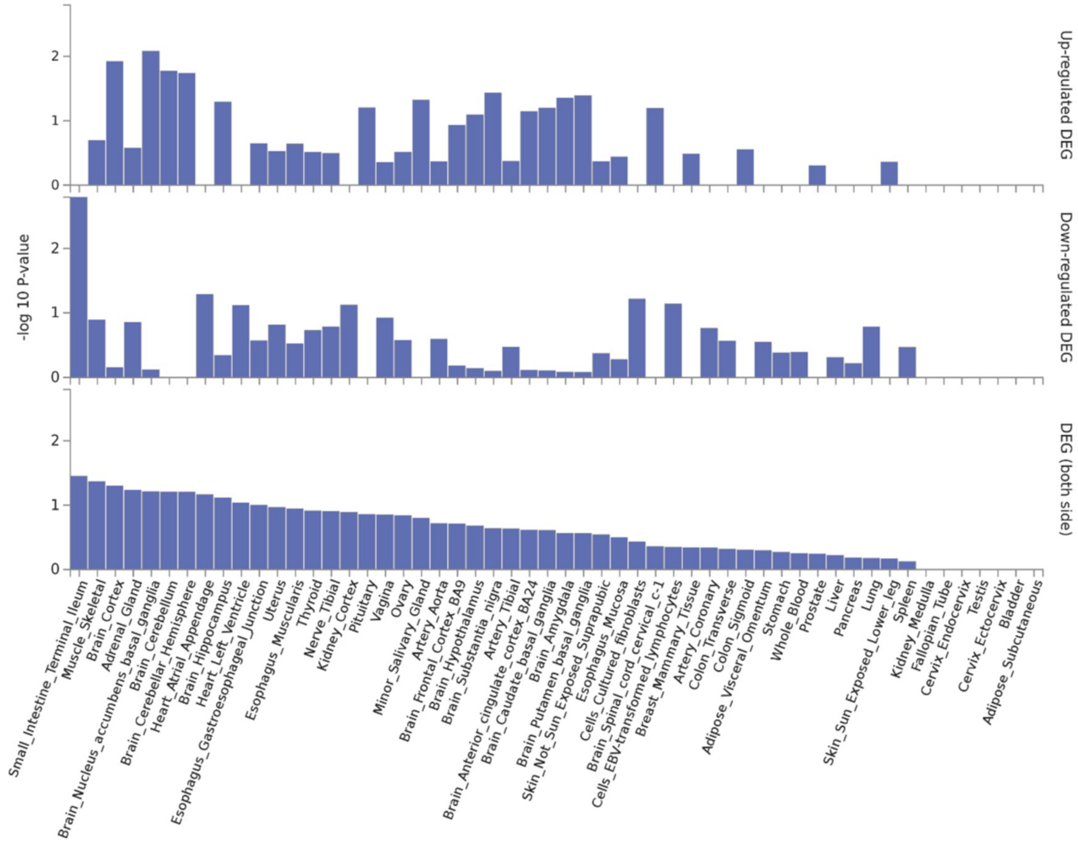

b

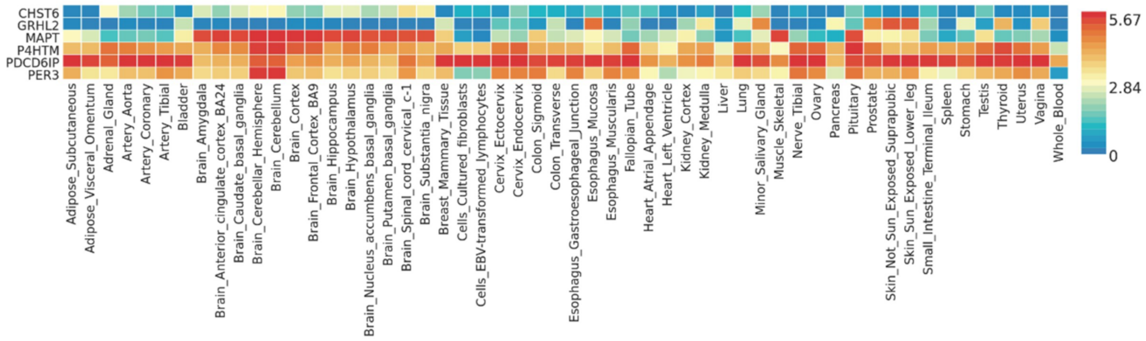

Supplementary Figure 2: Tissue expression and functional enrichment of seven top prioritized genes. a) The tissue expression of the 7 top prioritized genes, analyzed by Functional Mapping and Annotation (FUMA). All analyzed genes were compared using GTEx v8 tissue expression data against expression data from genes from the Database of Essential Genes (DEG). The top panel shows upregulated genes with comparison to DEG; the center panel shows downregulated genes compared to DEG, and the bottom panel shows both sides. Red bars are statistically significant. b) Expression heatmap of the top seven prioritized genes across all tissues in GTEx v8, as analyzed by Functional Mapping and Annotation (FUMA).

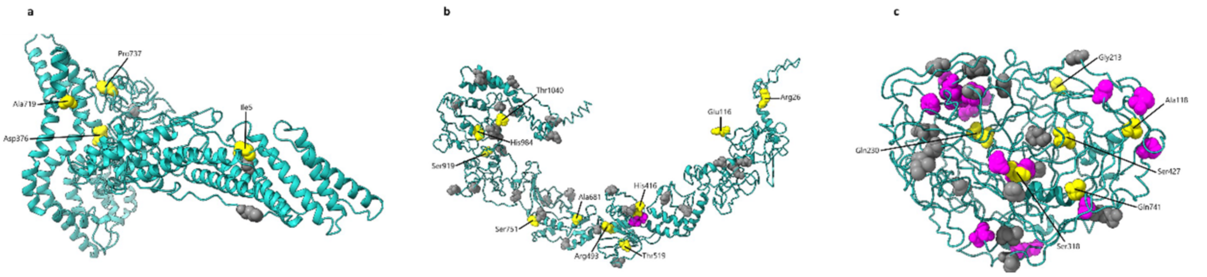

Supplementary Figure 3: Protein Models of PDCD6IP, PER3 and MAPT. 3D protein model of the candidates a) PDCD6IP, b) PER3, and c) MAPT. Variants that were predicted to be damaging or had high individual p-values are shown in yellow while other variants present in the analysis are shown in gray. ClinVar pathogenic variants are shown in magenta.

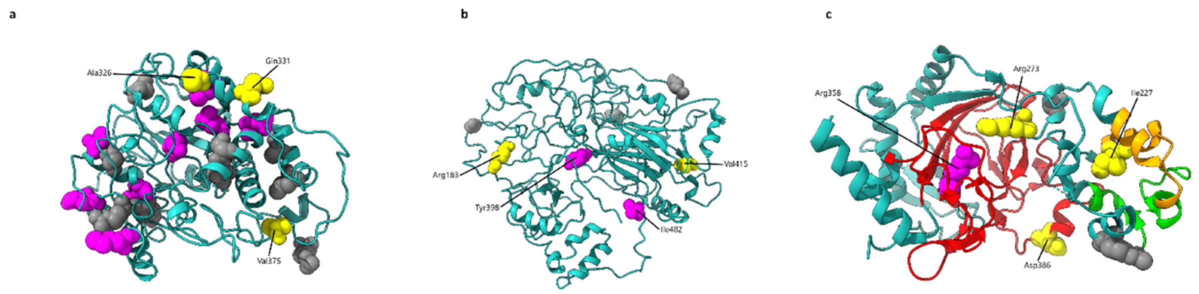

Supplementary Figure 4: Protein Model of CHST6, GRHL2, and P4HTM. 3D protein models of the candidates a) CHST6, b) GRHL2, and c) P4HTM. Variants that were predicted to be damaging or had high individual p-values are shown in yellow while other variants present in the analysis are shown in gray. ClinVar pathogenic variants are shown in magenta. In C) the Fe-dependent 2-OG dioxygenase domain is shown in red.

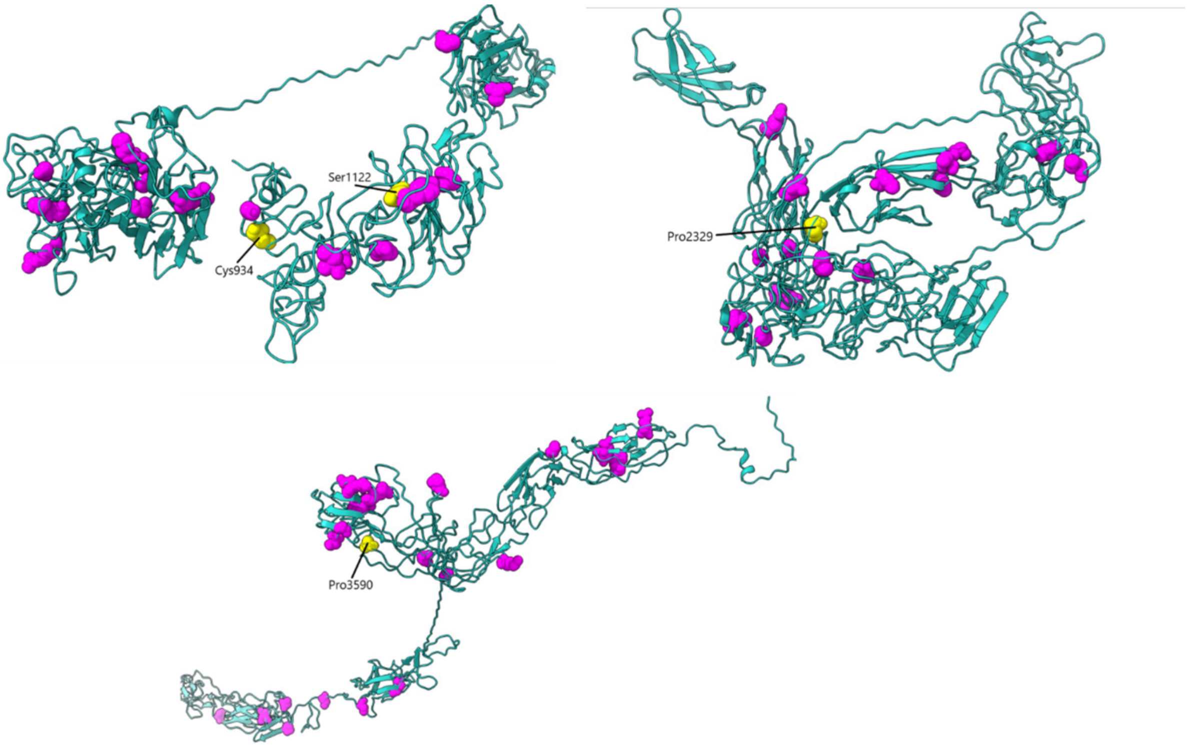

Supplementary Figure 5: Protein Model of USH2A. 3D protein model of the candidate USH2A. Variants that were predicted to be damaging or had high individual p-values are shown in yellow. ClinVar pathogenic variants are shown in magenta.

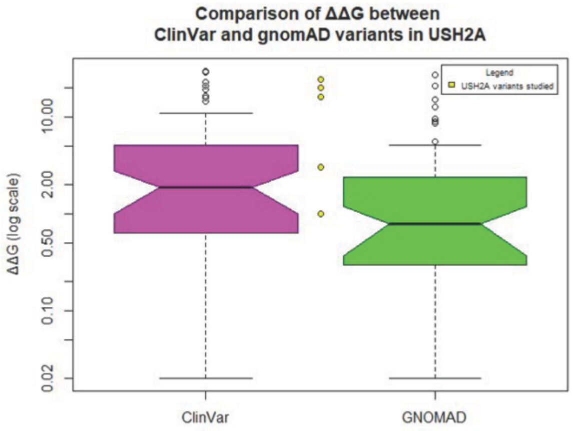

Supplementary Figure 6: Boxplot of ClinVar and gnomAD Variants in USH2A. Comparison between all USH2A ClinVar (n = 63) and gnomAD (n = 1870) variants in USH2A in terms of ΔΔG. The candidate variants identified in this study are shown in yellow. Whiskers represent the upper and lower bounds (standard errors) beyond which points are considered outliers. Middle line in both figures is considered the median

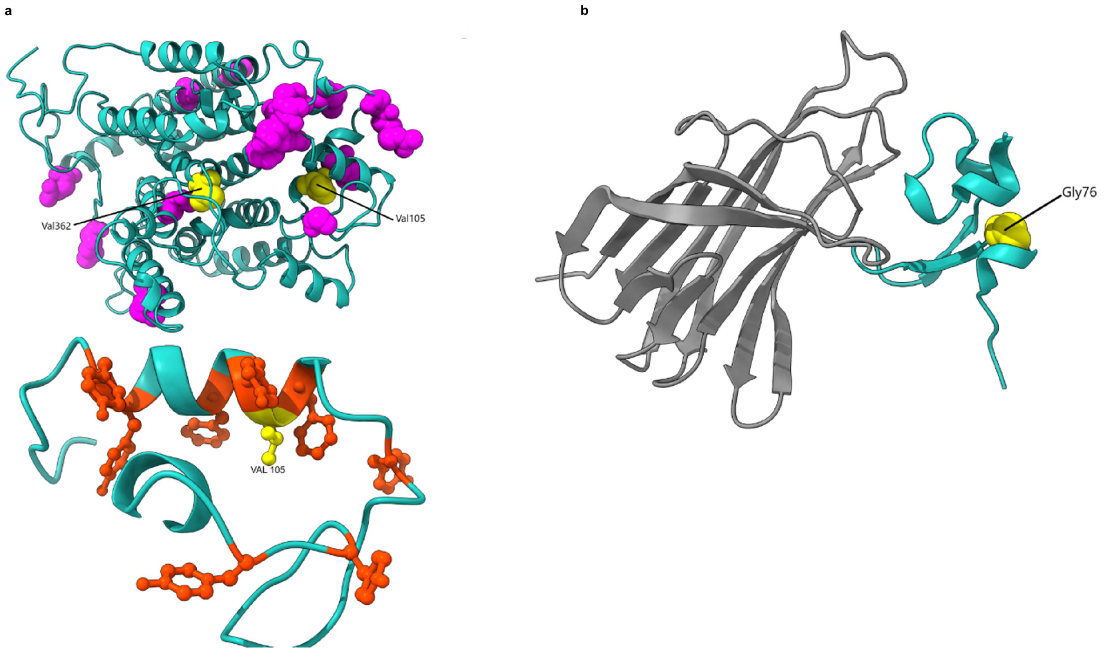

Supplementary Figure 7: Protein Models of ALG3 and TNFRSF13B. 3D protein models of the candidates a) ALG3 and b) TNFRSF13B. Variants that were predicted to be damaging or had high individual p-values are shown in yellow. ClinVar pathogenic variants are shown in magenta.

Supplementary References

1. Foong AW, Saw SM, Loo JL, Shen S, Loon SC, Rosman M, et al. Rationale and methodology for a population-based study of eye diseases in Malay people: The Singapore Malay eye study (SiMES). Ophthalmic Epidemiol. 2007;14(1):25-35.
2. Lavanya R, Jeganathan VS, Zheng Y, Raju P, Cheung N, Tai ES, et al. Methodology of the Singapore Indian Chinese Cohort (SICC) eye study: quantifying ethnic variations in the epidemiology of eye diseases in Asians. Ophthalmic Epidemiol. 2009;16(6):325-36.
3. Age-Related Eye Disease Study Research G. A randomized, placebo-controlled, clinical trial of high-dose supplementation with vitamins C and E and beta carotene for age-related cataract and vision loss: AREDS report no. 9. Arch Ophthalmol. 2001;119(10):1439-52.
4. Age-Related Eye Disease Study Research G. A randomized, placebo-controlled, clinical trial of high-dose supplementation with vitamins C and E, beta carotene, and zinc for age-related macular degeneration and vision loss: AREDS report no. 8. Arch Ophthalmol. 2001;119(10):1417-36.
5. Clemons TE, Chew EY, Bressler SB, McBee W, Age-Related Eye Disease Study Research G. National Eye Institute Visual Function Questionnaire in the Age-Related Eye Disease Study (AREDS): AREDS Report No. 10. Arch Ophthalmol. 2003;121(2):211-7.
6. Age-Related Eye Disease Study Research G. The Age-Related Eye Disease Study (AREDS): design implications. AREDS report no. 1. Control Clin Trials. 1999;20(6):573-600.
7. Ikram MA, Brusselle GGO, Murad SD, van Duijn CM, Franco OH, Goedegebure A, et al. The Rotterdam Study: 2018 update on objectives, design and main results. Eur J Epidemiol. 2017;32(9):807-50.
8. Pardo LM, MacKay I, Oostra B, van Duijn CM, Aulchenko YS. The effect of genetic drift in a young genetically isolated population. Ann Hum Genet. 2005;69(Pt 3):288-95.
9. Aulchenko YS, Heutink P, Mackay I, Bertoli-Avella AM, Pullen J, Vaessen N, et al. Linkage disequilibrium in young genetically isolated Dutch population. Eur J Hum Genet. 2004;12(7):527-34.
10. Milani L, Leitsalu L, Metspalu A. An epidemiological perspective of personalized medicine: the Estonian experience. J Intern Med. 2015;277(2):188-200.
11. Pärssinen O, Jauhonen HM, Kauppinen M, Kaprio J, Koskenvuo M, Rantanen T. Heritability of spherical equivalent: a population-based twin study among 63- to 76-year-old female twins. Ophthalmology. 2010;117(10):1908-11.
12. Biino G, Balduini CL, Casula L, Cavallo P, Vaccargiu S, Parracciani D, et al. Analysis of 12,517 inhabitants of a Sardinian geographic isolate reveals that predispositions to thrombocytopenia and thrombocytosis are inherited traits. Haematologica. 2011;96(1):96-101.
13. Vitart V, Bencić G, Hayward C, Herman JS, Huffman J, Campbell S, et al. Heritabilities of ocular biometrical traits in two croatian isolates with extended pedigrees. Invest Ophthalmol Vis Sci. 2010;51(2):737-43.
14. Spector TD, Williams FM. The UK Adult Twin Registry (TwinsUK). Twin Res Hum Genet. 2006;9(6):899-906.
15. Verdi S, Abbasian G, Bowyer RCE, Lachance G, Yarand D, Christofidou P, et al. TwinsUK: The UK Adult Twin Registry Update. Twin Res Hum Genet. 2019;22(6):523-9.
16. Linton KL, Klein BE, Klein R. The validity of self-reported and surrogate-reported cataract and age-related macular degeneration in the Beaver Dam Eye Study. American journal of epidemiology. 1991;134(12):1438-46.
17. Klein R, Klein BE, Linton KL, De Mets DL. The Beaver Dam Eye Study: visual acuity. Ophthalmology. 1991;98(8):1310-5.
18. Lee KE, Klein BE, Klein R, Fine JP. Aggregation of refractive error and 5-year changes in refractive error among families in the Beaver Dam Eye Study. Archives of ophthalmology (Chicago, Ill : 1960). 2001;119(11):1679-85.
19. Klein AP, Duggal P, Lee KE, Cheng CY, Klein R, Bailey-Wilson JE, et al. Linkage analysis of quantitative refraction and refractive errors in the Beaver Dam Eye Study. Investigative ophthalmology & visual science. 2011;52(8):5220-5.

20. Day N, Oakes S, Luben R, Khaw KT, Bingham S, Welch A, et al. EPIC-Norfolk: study design and characteristics of the cohort. European Prospective Investigation of Cancer. Br J Cancer. 1999;80 Suppl 1:95-103.
21. Morava E, Wosik HN, Sykut-Cegielska J, Adamowicz M, Guillard M, Wevers RA, et al. Ophthalmological abnormalities in children with congenital disorders of glycosylation type I. British journal of ophthalmology. 2009;93(3):350-4.
22. Park PJ, Shukla D. Role of heparan sulfate in ocular diseases. Experimental eye research. 2013;110:1-9.
