# Supplementary material for: Rare variant analyses across multiethnic cohorts identify novel genes for refractive error
Source: Commun Biol. 2023 Jan 3;6:6. doi: 10.1038/s42003-022-04323-7 (PMC9810640; doi:10.1038/s42003-022-04323-7)
Supplement: Supplementary file 3 — Description of Additional Supplemenary Files [file 42003_2022_4323_MOESM3_ESM.pdf]

Description of Additional Supplementary Files

File name: Supplementary Data 1-12

Description:

Supplementary Data 1: Description of Cohorts. The ethnicities, nationalities, individual number and mean spherical equivalent (SER) from all the analyzed cohorts

Supplementary Data 2: Polygenic Risk Score Calculation values. The polygenic risk score calculations for each cohort

Supplementary Data 3: Explained variance of top individual genes using EMMAX-VT with and without PRS covariates.

Supplementary Data 4: Explained variance of top individual genes using EMMAX-CMC with and without PRS covariates.

Supplementary Data 5: All Unique Genome-wide significant Genes. The list of all unique genome-wide significant genes found through all analyses.

Supplementary Data 6: Multiethnic meta-analysis genome-wide significant p-values for EMMAX-VT. The p-values for all genome-wide significant genes in the meta-analysis for all cohorts.

Supplementary Data 7: Multiethnic meta-analysis genome-wide significant p-values for EMMAX-CMC. The p-values for all genome-wide significant genes in the meta-analysis for all cohorts.

Supplementary Data 8: Multiethnic meta-analysis genome-wide significant p-values for ACAT. The p-values for all genome-wide significant genes in the meta-analysis for all cohorts.

Supplementary Data 9: All multiethnic meta-analysis p-values for EMMAX-VT. P-values for all genes in the meta-analysis.

Supplementary Data 10: All multiethnic meta-analysis p-values for EMMAX-CMC. P-values for all genes in the meta-analysis.

Supplementary Data 11: All multiethnic meta-analysis p-values for ACAT. P-values for all genes in the meta-analysis.

Supplementary Data 12: Indo-European meta-analysis genome-wide significant p-values for EMMAX-VT.

File name: Supplementary Data 13-25

Description:

Supplementary Data 13: Indo-European meta-analysis genome-wide significant p-values for EMMAX-CMC.

Supplementary Data 14: Indo-European meta-analysis genome-wide significant p-values for ACAT.

Supplementary Data 15: All meta-analysis p-values using Indo-European cohorts for EMMAX-VT.

Supplementary Data 16: All meta-analysis p-values using Indo-European cohorts only for EMMAX-CMC.

Supplementary Data 17: All meta-analysis p-values using all Indo-European cohorts for ACAT.

Supplementary Data 18: Genome-wide significant p-values in EACC cohort for EMMAX-VT.

Supplementary Data 19: Genome-wide significant p-values in EACC cohort for EMMAX-CMC.

Supplementary Table 20: Genome-wide significant meta-analysis p-values using EACC cohorts for ACAT.

Supplementary Data 21: Genes significant in one cohort but no rare variants present in other cohorts.

Supplementary Data 22: P-values of Replicated Genome-wide significant genes using UK BioBank.

Supplementary Data 23: Upstream Regulators of the 129 Associated Genes. The upstream regulators of all 129 unique genes as provided by IPA.

Supplementary Data 24: Causal Network Analysis of 129 Associated Genes. The causal network analysis all 129 unique genes as provided by IPA.

Supplementary Data 25: Diseases and Functional Analysis of 129 Associated Genes. The disease and functional analysis of all 129 unique genes as provided by IPA.

File name: Supplementary Data 26

Description: Contains six individual tables (26A-26F).

Supplementary Data 26a - Results of EMMAX VT analysis using all cohorts
Supplementary Data 26b - Results of EMMAX CMC, VT and ACAT analysis using different cohorts
Supplementary Data 26c - Results of EMMAX analysis using East-Asian combined cohort (EACC) only
Supplementary Data 26d - Look up of genes significant in EMMAX VT analysis using all cohorts
Supplementary Data 26e - Look up of genes significant in EMMAX CMC, VT and ACAT analyses using different cohorts
Supplementary Data 26f - Look up of genes significant in EMMAX analysis using East-Asian combined cohort (EACC) only

File name: Supplementary Data 27-34

Description:
Supplementary Data 27: Average SER of Cases/Controls of Variants in Prioritized Genes. Contains the average SER for both cases and controls of variants in prioritized genes.

Supplementary Data 28: EMMAX-CMC Test Betas for Prioritized Genes.

Supplementary Data 29: Upstream Regulators of the Seven Top Prioritized Genes. The upstream regulators of 7 prioritized genes as provided by IPA.

Supplementary Data 30: Causal Network Analysis of Seven Top Prioritized Genes. The causal network analysis of 7 prioritized genes as provided by IPA.

Supplementary Table 31: Diseases and Functional Analysis of Seven Top Prioritized Genes. The disease and functional analysis of 7 prioritized genes as provided by IPA.

Supplementary Data 32: Free Energy Difference (ΔΔG) caused by Mutations in Potential Causal Variants in Prioritized Genes.

Supplementary Data 33: Potential Causal Variants in Genome-wide Significant Genes. Protein prediction for the potential causal variants in the genome-wide significant genes.

Supplementary Data 34: Free Energy Difference (ΔΔG) caused by Mutations in Potential Causal Variants in Additional Candidate Genes.

File name: Supplementary Software 1

Description: File that contains the R scripts used in the analyses presented in this paper.
