# Supplementary material for: Rare variant analyses across multiethnic cohorts identify novel genes for refractive error
Source: Commun Biol. 2023 Jan 3;6:6. doi: 10.1038/s42003-022-04323-7 (PMC9810640; doi:10.1038/s42003-022-04323-7)
Supplement: Supplementary file 8 — Supplementary Software 1 [file 42003_2022_4323_MOESM8_ESM.docx]

**Script to Analyze IBD**

declare -a sites=("AREDS")

for element in ${sites[@]}

do

plink1.9 --bfile $element-cleanedAutosomes-hwe-preIBD --missing --out $element-cleanedAutosomes-hwe-preIBD

done

R CMD BATCH --no-save analyze-ibd.ra

##Anthony Musolf

##March 5, 2018

##analyze-ibd.r

##This script identifies the twins/duplicates in a *.genome file. It also identifies any people that may be related to too many other individuals in the data set

sites = c("AREDS")

for (i in 1:length(sites)) {

data = matrix(scan(paste(sites[i], "-cleanedAutosomes-common.genome", sep = ""), what = "", sep = ""), ncol = 14, byrow = T)

data = data[-1,]

dataL = scan(paste(sites[i], "-cleanedAutosomes-common.genome", sep = ""), what = "", sep = "\n")

dataL = dataL[-1]

fam1 = data[,1]

ind1 = data[,2]

fam2 = data[,3]

ind2 = data[,4]

z0 = as.numeric(data[,7])

z1 = as.numeric(data[,8])

##Find Twins

mrk1 = which(z0 < 0.05)

mrk2 = which(z1 < 0.05)

u12 = which(mrk1 %in% mrk2)

aMrk1 = mrk1[u12]

fin = dataL[aMrk1]

if (length(fin) > 0) {

write.table(fin, paste("twins-full-IBD-", sites[i], ".txt", sep = ""), quote=F,row.names=F,col.names=F,sep="\t")

} else {

fin = "NA"

write.table(fin, paste("twins-full-IBD-", sites[i], ".txt", sep = ""), quote=F,row.names=F,col.names=F,sep="\t")

}

ibd2 = matrix(scan(paste("twins-full-IBD-", sites[i], ".txt", sep = ""), what = "", sep = ""), ncol = 14, byrow = T)

if (fin[1] == "NA") {

twins = c("NA", "NA")

twins = matrix(twins, ncol = 2, byrow = T)

write.table(twins, paste("twins-", sites[i], ".txt", sep = ""), quote=F,row.names=F,col.names=F,sep="\t")

} else {

imiss = matrix(scan(paste(sites[i], "-cleanedAutosomes-hwe-preIBD.imiss", sep = ""), what = "", sep = ""), ncol = 6, byrow = T)

twins = c()

for (j in 1:length(ibd2[,1])) {

p1 = ibd2[j,2]

p2 = ibd2[j,4]

q1 = match(p1, imiss[,2])

q2 = match(p2, imiss[,2])

if (imiss[q1,6] >= imiss[q2,6]) {

twins = c(twins, imiss[q1,1], imiss[q1,2])

} else {

twins = c(twins, imiss[q2,1], imiss[q2,2])

}

}

twins = matrix(twins, ncol = 2, byrow = T)

write.table(twins, paste("twins-", sites[i], ".txt", sep = ""), quote=F,row.names=F,col.names=F,sep="\t")

}

##Find people that may be related to too many other people

high = which(z0 < 0.75)

hFam1 = fam1[high]

hInd1 = ind1[high]

hFam2 = fam2[high]

hInd2 = ind2[high]

allHinds = c(hInd1, hInd2)

tallHinds = table(allHinds)

all = which(tallHinds > 10)

if (length(all) > 0) {

write.table(tallHinds[all], paste("bad-IBD-counts-", sites[i], ".txt", sep = ""), quote=F,row.names=T,col.names=F,sep="\t")

data2 = matrix(scan(paste("bad-IBD-counts-", sites[i], ".txt", sep = ""), what = "", sep = ""), ncol = 2, byrow = T)

iids = data2[,1]

fams = matrix(scan(paste(sites[i], "-cleanedAutosomes-common.fam", sep = ""), what = "", sep = ""), ncol = 6, byrow = T)

lines = c()

for (j in 1:length(iids)) {

mrk = match(iids[j], fams[,2])

lines = c(lines, mrk)

}

allfam = fams[,1]

fids = allfam[lines]

fids = matrix(allfam[lines], ncol = 1, byrow = T)

iids = matrix(iids, ncol = 1, byrow = T)

fin = cbind(fids, iids)

write.table(fin, paste("bad-IBD-", sites[i], ".txt", sep = ""), quote=F,row.names=F,col.names=F,sep="\t")

} else {

fin = "NA"

write.table(fin, paste("bad-IBD-counts-", sites[i], ".txt", sep = ""), quote=F,row.names=T,col.names=F,sep="\t")

write.table(fin, paste("bad-IBD-", sites[i], ".txt", sep = ""), quote=F,row.names=F,col.names=F,sep="\t")

}

}

**Script to Perform Quality Control**

declare -a sites=("AREDS")

for element in ${sites[@]}

do

mv test.bim $element.bim

plink1.9 --bfile $element-cleanedAutosomes-hwe-preIBD --remove twins-$element.txt --make-bed --out $element-cleanedAutosomes-noTwins

plink1.9 --bfile $element-cleanedAutosomes-noTwins --geno 0.01 --maf 0.000001 --make-bed --out $element-cleanedAutosomes-r3

lines=$(wc -l < $element-cleanedAutosomes-r3.bim)

bonf=$(echo "0.05/$lines" | bc -l)

plink1.9 --bfile $element-cleanedAutosomes-r3 --hwe $bonf --make-bed --out ../final-related/$element-cleanedAutosomes-related-final

done

**Script to Calculate IBD**

declare -a sites=("AREDS")

for element in ${sites[@]}

do

plink1.9 --bfile $element-cleanedAutosomes-hwe-preIBD --maf 0.05 --make-bed --out $element-cleanedAutosomes-common

plink1.9 --bfile $element-cleanedAutosomes-common --genome --out $element-cleanedAutosomes-common

done

**Script to Convert Plink to VCF files**

declare -a files=("AREDS")

for element in ${files[@]}

do

pseq eye new-project

pseq eye load-plink -f $element-cleanedAutosomes-related-final-pheno --id eye1

pseq eye write-vcf > eye.vcf

sed 's/chr//g' eye.vcf > $element.vcf

bgzip $element.vcf

tabix -p vcf -f $element.vcf.gz

done

**Script to Extract Autosomes**

sites = c("AREDS")

for (i in 1:length(sites)) {

curSite = sites[i]

data = scan(paste(curSite, "-clean.bim", sep = ""), what = "", sep = "")

data = matrix(data, ncol = 6, byrow= T)

chr = as.numeric(data[,1])

snp = data[,2]

#noPo = which(chr == 0)

rest = which(chr >=23)

nonAuto = rest

#nonAuto = c(noPo, rest)

all = snp[nonAuto]

write.table(all, paste(curSite, "-nonAutosomes.txt", sep = ""), quote=F,row.names=F,col.names=F,sep="\t")

}

**Script to Make Kinship Matrix**

declare -a files=("AREDS")

for element in ${files[@]}

do

epacts make-kin --vcf $element.vcf.gz --min-maf 0.01 --min-callrate 0.95 --out $element.kinf --run 2

epacts-anno --in $element.vcf.gz --ref /hdata/musolfam/longka_chiari_20161215/chiari/scripts/human_g1k_v37.fasta --out $element.anno.vcf.gz

epacts make-group --vcf $element.anno.vcf.gz --out $element-genes.grp --nonsyn

epacts make-group --vcf $element.anno.vcf.gz --out $element-genesWS.grp --type Nonsynonymous --type Stop_Gain --type Essential_Splice_Site --type Start_Loss --type Stop_Loss --type Normal_Splice_Site --type Synonymous

done

mkdir RE/

mkdir RE/gene-nonsynonymousOnly

mkdir RE/gene-nonsynonymousOnly/CMC

mkdir RE/gene-nonsynonymousOnly/VT

mkdir RE/gene-withSynon

mkdir RE/gene-withSynon/CMC

mkdir RE/gene-withSynon/VT

mkdir RE/single-variant

**Script to Run EMMAX-CMC**

declare -a files=("AREDS")

for element in ${files[@]}

do

epacts group --groupf $element-genesWS.grp --vcf $element.vcf.gz --ped $element.ped --max-maf 0.05 --kin $element.kinf --pheno PHE --cov SEX --cov AGE --cov EDU --test emmaxCMC --out gene-withSynon/CMC/$element-CMC-ASE-0.05 --run 2

epacts group --groupf $element-genesWS.grp --vcf $element.vcf.gz --ped $element.ped --max-maf 0.01 --kin $element.kinf --pheno PHE --cov SEX --cov AGE --cov EDU --test emmaxCMC --out gene-withSynon/CMC/$element-CMC-ASE-0.01 --run 2

epacts group --groupf $element-genesWS.grp --vcf $element.vcf.gz --ped $element.ped --max-maf 0.05 --kin $element.kinf --pheno PHE --cov SEX --cov AGE --test emmaxCMC --out gene-withSynon/CMC/$element-CMC-AS-0.05 --run 2

epacts group --groupf $element-genesWS.grp --vcf $element.vcf.gz --ped $element.ped --max-maf 0.01 --kin $element.kinf --pheno PHE --cov SEX --cov AGE --test emmaxCMC --out gene-withSynon/CMC/$element-CMC-AS-0.01 --run 2

epacts group --groupf $element-genesWS.grp --vcf $element.vcf.gz --ped $element.ped --max-maf 0.05 --kin $element.kinf --pheno PHE --test emmaxCMC --out gene-withSynon/CMC/$element-CMC-noCov-0.05 --run 2

epacts group --groupf $element-genesWS.grp --vcf $element.vcf.gz --ped $element.ped --max-maf 0.01 --kin $element.kinf --pheno PHE --test emmaxCMC --out gene-withSynon/CMC/$element-CMC-noCov-0.01 --run 2

done

**Script to Run EMMAX-VT**

declare -a files=("AREDS")

for element in ${files[@]}

do

epacts group --groupf $element-genesWS.grp --vcf $element.vcf.gz --ped $element.ped --max-maf 0.05 --kin $element.kinf --pheno PHE --cov SEX --cov AGE --cov EDU --test emmaxVT --out gene-withSynon/VT/$element-VT-ASE-0.05 --run 2

epacts group --groupf $element-genesWS.grp --vcf $element.vcf.gz --ped $element.ped --max-maf 0.01 --kin $element.kinf --pheno PHE --cov SEX --cov AGE --cov EDU --test emmaxVT --out gene-withSynon/VT/$element-VT-ASE-0.01 --run 2

epacts group --groupf $element-genesWS.grp --vcf $element.vcf.gz --ped $element.ped --max-maf 0.05 --kin $element.kinf --pheno PHE --cov SEX --cov AGE --test emmaxVT --out gene-withSynon/VT/$element-VT-AS-0.05 --run 2

epacts group --groupf $element-genesWS.grp --vcf $element.vcf.gz --ped $element.ped --max-maf 0.01 --kin $element.kinf --pheno PHE --cov SEX --cov AGE --test emmaxVT --out gene-withSynon/VT/$element-VT-AS-0.01 --run 2

epacts group --groupf $element-genesWS.grp --vcf $element.vcf.gz --ped $element.ped --max-maf 0.05 --kin $element.kinf --pheno PHE --test emmaxVT --out gene-withSynon/VT/$element-noCov-0.05 --run 2

epacts group --groupf $element-genesWS.grp --vcf $element.vcf.gz --ped $element.ped --max-maf 0.01 --kin $element.kinf --pheno PHE --test emmaxVT --out gene-withSynon/VT/$element-noCov-0.01 --run 2

done

**Script to Run EMMAX Single Variant**

mkdir single-variant

declare -a files=("AREDS")

for element in ${files[@]}

do

epacts single --vcf $element.vcf.gz --ped $element.ped --min-mac 3 --kin $element.kinf --pheno PHE --cov SEX --cov AGE --cov EDU --test q.emmax --out single-variant/$element-ASE --run 2

epacts single --vcf $element.vcf.gz --ped $element.ped --min-mac 3 --kin $element.kinf --pheno PHE --cov SEX --cov AGE --test q.emmax --out single-variant/$element-AS --run 2

epacts single --vcf $element.vcf.gz --ped $element.ped --min-mac 3 --kin $element.kinf --pheno PHE --test q.emmax --out single-variant/$element-noCov --run 2

done
