# Supplementary material for: Rare variant analyses across multiethnic cohorts identify novel genes for refractive error
Source: Commun Biol. 2023 Jan 3;6:6. doi: 10.1038/s42003-022-04323-7 (PMC9810640; doi:10.1038/s42003-022-04323-7)
Supplement: Supplementary file 9 — Reporting Summary [file 42003_2022_4323_MOESM9_ESM.pdf]

nature research

Corresponding author(s): Joan E. Bailey-Wilson and Caroline Klaver

Last updated by author(s): Nov 2, 2022

Reporting Summary

Nature Research wishes to improve the reproducibility of the work that we publish. This form provides structure for consistency and transparency in reporting. For further information on Nature Research policies, see our Editorial Policies and the Editorial Policy Checklist.

Statistics

For all statistical analyses, confirm that the following items are present in the figure legend, table legend, main text, or Methods section.

n/a	Confirmed	
☐	☒	The exact sample size (*n*) for each experimental group/condition, given as a discrete number and unit of measurement
☒	☐	A statement on whether measurements were taken from distinct samples or whether the same sample was measured repeatedly
☐	☒	The statistical test(s) used AND whether they are one- or two-sided<br>*Only common tests should be described solely by name; describe more complex techniques in the Methods section.*
☐	☒	A description of all covariates tested
☐	☒	A description of any assumptions or corrections, such as tests of normality and adjustment for multiple comparisons
☐	☒	A full description of the statistical parameters including central tendency (e.g. means) or other basic estimates (e.g. regression coefficient) AND variation (e.g. standard deviation) or associated estimates of uncertainty (e.g. confidence intervals)
☐	☒	For null hypothesis testing, the test statistic (e.g. *F*, *t*, *r*) with confidence intervals, effect sizes, degrees of freedom and *P* value noted<br>*Give P values as exact values whenever suitable.*
☒	☐	For Bayesian analysis, information on the choice of priors and Markov chain Monte Carlo settings
☒	☐	For hierarchical and complex designs, identification of the appropriate level for tests and full reporting of outcomes
☐	☒	Estimates of effect sizes (e.g. Cohen's *d*, Pearson's *r*), indicating how they were calculated

Our web collection on statistics for biologists contains articles on many of the points above.

Software and code

Policy information about availability of computer code

Data collection	no such sofrware was used
Data analysis	Freely available data analysis software is described and referenced in the methods section

For manuscripts utilizing custom algorithms or software that are central to the research but not yet described in published literature, software must be made available to editors and reviewers. We strongly encourage code deposition in a community repository (e.g. GitHub). See the Nature Research guidelines for submitting code & software for further information.

Data

Policy information about availability of data

All manuscripts must include a data availability statement. This statement should provide the following information, where applicable:

- Accession codes, unique identifiers, or web links for publicly available datasets
- A list of figures that have associated raw data
- A description of any restrictions on data availability

The data that support the findings of this study are not publicly available due to information that could compromise research participant privacy and/or consent. European Union data privacy rulings currently forbid sharing of genomic data outside the EU and several of the parent studies have additional restrictions to protect the privacy of the study participants. Data were used here under data use agreements with each participating study. Data may be available by request from the individual participating studies if all regulatory conditions are met.

Field-specific reporting

Please select the one below that is the best fit for your research. If you are not sure, read the appropriate sections before making your selection.

☒ Life sciences ☐ Behavioural & social sciences ☐ Ecological, evolutionary & environmental sciences

For a reference copy of the document with all sections, see nature.com/documents/nr-reporting-summary-flat.pdf

Life sciences study design

All studies must disclose on these points even when the disclosure is negative.

Sample size	Sample sizes were chosen by rerquesting participation from all CREAM consortium studies that had genotyped the exome chip on study participants. All available samples were included. Sample sizes are similar to many prior successful genome-wide association studies.
Data exclusions	Individuals who had undergone procedures that could alter refraction, e.g., cataract surgery, laser refractive error procedures, retinal detachment surgery, and other ophthalmic conditions that may influence refraction were excluded from these analyses. This is a standard exclusion in all previous CREAM analyses of refractive error. Any individual not genotyped at 99% of all variants was removed and any variant not genotyped at 99% was also removed. Variants with a HWE p-value less than a Bonferroni-corrected p-value (defined as 0.05 / total number of variants in the dataset) were also excluded. We also checked for batch effects and calculated the identity-by-descent (IBD) value of all individuals in the cohort, removing duplicates and twins. These are standard QC steps and were part of the original study design.
Replication	The most powerful meta-analyses in this study used all the samples so that replication was attempted using results from the UK Biobank. The genes that replicated are reported in this manuscript. Additionally, some genes were genome-wide significant in individual cohorts and in that case we determined which associated genes were replicated in one or more of the other cohorts. Many of the genome-wide significantly associated genes were not replicated. In some situations, this was because none of the other cohorts had ANY rare variants in those genes so replication was not possible. Of the remaining unreplicated associations, some of them are almost certainly false positive results but some may be unreplicated because DIFFERENT rare variants were present in the same genes in the different cohorts, with damaging rare variants being present in the cohorts with significant association and non-damaging rare variants being present in the cohorts without association. Future studies are needed to resolve this question.
Randomization	Randomization is not applicable to this sort of genome-wide association study since under the null hypothesis, the independent variables (genotypes) are not correlated with covariates that affect refractive error such as age and time spent outdoors.
Blinding	There was no group allocation. This is an analysis of a quantitative trait in population-based studies where individuals were not recruited into the studies on the basis of the trait, refractive error.

Reporting for specific materials, systems and methods

We require information from authors about some types of materials, experimental systems and methods used in many studies. Here, indicate whether each material, system or method listed is relevant to your study. If you are not sure if a list item applies to your research, read the appropriate section before selecting a response.

Materials & experimental systems

n/a	Involved in the study	
☒	☐	Antibodies
☒	☐	Eukaryotic cell lines
☒	☐	Palaeontology and archaeology
☒	☐	Animals and other organisms
☐	☒	Human research participants
☒	☐	Clinical data
☒	☐	Dual use research of concern

Methods

n/a	Involved in the study	
☒	☐	ChIP-seq
☒	☐	Flow cytometry
☒	☐	MRI-based neuroimaging

Human research participants

Policy information about studies involving human research participants

Population characteristics	This is a meta-analysis of multiple previously published large population-based studies of both men and women, all adults over age 18 with most in middle age. Enrollment was not based on refractive error, no treatments are given as part of this study and no treatments or clinical outcomes are studied. This study is of refractive error as measured upon enrollment into these population-based studies.
Recruitment	All of these studies were population-based large epidemiological studies. Since none of them were enrolling based on refractive error phenotype, there should be no self-selection bias.
Ethics oversight	The IRB of the National Institutes of Health considered this study "Not Human Subjects" since only deidentified data were

Ethics oversight	shared by the participating studies with the analysts performing the statistical analyses in this study. Several studies only shared analysis p-values rather than deidentified data due to their IRB restrictions. The original data collection, genotyping and genetic analyses were approved individually by the IRBs of each of the parent studies. More details have been previously published about all of these studies and extensive detail, referring to these publications is given in the manuscript and in the Supplemental Methods.

Note that full information on the approval of the study protocol must also be provided in the manuscript.
